# NuMA promotes homologous recombination repair by regulating the accumulation of the ISWI ATPase SNF2h at DNA breaks

**DOI:** 10.1093/nar/gku296

**Published:** 2014-04-20

**Authors:** Pierre-Alexandre Vidi, Jing Liu, Daniela Salles, Swaathi Jayaraman, George Dorfman, Matthew Gray, Patricia Abad, Prabhas V. Moghe, Joseph M. Irudayaraj, Lisa Wiesmüller, Sophie A. Lelièvre

**Affiliations:** 1 Department of Basic Medical Sciences, Purdue University, West Lafayette, IN 47907, USA; 2 Department of Agricultural and Biological Engineering, Purdue University, West Lafayette, IN 47907, USA; 3 Department of Obstetrics and Gynecology, University of Ulm, Prittwitzstrasse 43, D-89075 Ulm, Germany; 4 Department of Biomedical Engineering, and Chemical & Biochemical Engineering, Rutgers University, Piscataway, NJ 08854, USA; 5 Center for Cancer Research, Purdue University, West Lafayette, IN 47907, USA

## Abstract

Chromatin remodeling factors play an active role in the DNA damage response by shaping chromatin to facilitate the repair process. The spatiotemporal regulation of these factors is key to their function, yet poorly understood. We report that the structural nuclear protein NuMA accumulates at sites of DNA damage in a poly[ADP-ribose]ylation-dependent manner and functionally interacts with the ISWI ATPase SNF2h/SMARCA5, a chromatin remodeler that facilitates DNA repair. NuMA coimmunoprecipitates with SNF2h, regulates its diffusion in the nucleoplasm and controls its accumulation at DNA breaks. Consistent with NuMA enabling SNF2h function, cells with silenced NuMA exhibit reduced chromatin decompaction after DNA cleavage, lesser focal recruitment of homologous recombination repair factors, impaired DNA double-strand break repair in chromosomal (but not in episomal) contexts and increased sensitivity to DNA cross-linking agents. These findings reveal a structural basis for the orchestration of chromatin remodeling whereby a scaffold protein promotes genome maintenance by directing a remodeler to DNA breaks.

## INTRODUCTION

DNA double-strand breaks (DSBs) are common and highly deleterious lesions in eukaryotic cells that can lead to mutations and chromosomal translocations linked with cancer development. DSBs are processed either by nonhomologous end-joining (NHEJ) or by homology-dependent repair pathways (1). Recombination repair by single strand annealing between repetitive DNA sequences and NHEJ are inherently mutagenic mechanisms whereas canonical homologous recombination repair (HR) is an error-free pathway that restores the genetic information at the damage site using the sister chromatid as a template. These pathways operate in the context of a complex, hierarchically organized chromatin environment that restricts the accessibility of repair factors to DNA lesions.

Different classes of adenosine triphosphate (ATP)-dependent chromatin remodeling complexes facilitate DNA repair, presumably by altering chromatin structure and nucleosome positioning at or near DNA breaks (2–4). Much attention has been devoted to the ISWI ATPase SNF2h/SMARCA5, a chromatin remodeler that rapidly accumulates at sites of DNA damage and is essential for the repair of DSBs (5–7). Yet, the mechanisms underlying targeting and retention of SNF2h at damaged chromatin remain poorly understood.

The timely recruitment of factors involved in the response to DNA damage is paramount for DNA repair. An attractive hypothesis is that the spatiotemporal coordination of the DNA damage response (DDR) involves structural elements of the cell nucleus, in particular proteins with scaffolding domains. Indeed, cells derived from patients with laminopathies express truncated or unprocessed variants of lamin A and have a higher sensitivity to genotoxic agents as well as constitutively elevated DNA damage (8). In these cells, the nucleotide excision repair factor XPA atypically accumulates at DSBs and the recruitment of repair factors 53BP1 and RAD51 is compromised (8,9). The mechanism linking A-type lamins and DNA repair involves the control of expression of 53BP1, BRCA1 and RAD51 (10). A second example of a structural nuclear protein involved in the DDR is nonerythroid alpha spectrin that accumulates at DNA lesions induced by cross-linking agents and mediates the recruitment of the nucleotide excision repair protein XPF (11). None of these actions, however, encompasses the chromatin remodeling aspect of the DDR. The nuclear mitotic apparatus protein (NuMA), an abundant coiled-coil protein related to lamins (12,13), has an unresolved impact on genome integrity. Proteomic studies have revealed NuMA phosphorylation after cell exposure to UV, ionizing radiations (IR) and chemotherapeutic drugs (14–17), and spatial rearrangement of NuMA was measured in response to DNA damage (18). We have also established that NuMA influences higher-order chromatin organization (i.e. the compartmentalization of euchromatin and heterochromatin) during mammary epithelial cell differentiation (19).

The potential connection between NuMA and the chromatin response during the DDR was investigated. We demonstrate that NuMA interacts with the WICH (WSTF-ISWI chromatin remodeling) complex and accumulates at DNA breaks. It functions by specifically controlling SNF2h presence at DNA damage sites in a poly[ADP-ribose]ylation context, and consequently promotes chromatin remodeling and Rad51-dependent HR repair activity. These findings establish the regulation of a chromatin remodeler by a structural nuclear protein.

## MATERIALS AND METHODS

### Cells

HMT-3522 S1 non-neoplastic breast epithelial cells and HMT-3522 T4-2 breast cancer cells were cultured in H14 medium (20). Breast cancer MCF-7 and osteosarcoma U2OS cells were cultured in Dulbecco's modified Eagle's medium supplemented with 10% fetal bovine serum. K562 erythroleukemic cells and lymphoblastoid lines with wild-type ATM (HA169 and TK6) or ATM-null ATM mutation (HA433) were cultured as described (21,22). A Gammacell 220 irradiator (Nordion) was used as the source of IRs.

### Protein fractionation, immunoprecipitation and western blot analysis

To resolve nuclear multiprotein complexes, nuclear extracts from S1 cells (23) were loaded onto a 10–40% sucrose gradient and ultracentrifuged for 40 h at 4°C and 214 000 g. Fractions of equal volumes were precipitated with trichloroacetic acid and analyzed with the pellet (insoluble fraction) by western blot. In immunoprecipitation (IP) experiments, nuclear extracts (1 mg) were incubated with antibodies overnight at 4°C and further processed using the Universal Magnetic Co-IP kit (Active Motif) according to the manufacturer's instructions. Antibodies used for immunoblotting were: 53BP1 (Abcam, Ab36823, 1 μg/ml), BRCA1 (Calbiochem, MS110, 5 μg/ml), BRG1 (Milipore, 07–478, 1:10000), DNA-PKcs (Abcam, clone 18–2, 2 μg/ml), γH2AX (Ser139; Millipore, clone JBW301, 1 μg/ml), Histone H2B (Abcam, Ab1790, 0.1 μg/ml), lamin B (Abcam, Ab16048, 60 ng/ml), NuMA (B1C11, 1:2, a gift from Dr Jeffrey Nickerson, UMass, Worcester, USA), PAR (Trevigen, 4336-APC-050, 1:1000), phospho-NuMA (Cell Signaling, 1:1000), SNF2h (Abcam, Ab3749, 1 μg/ml), RAD51 (Abcam, Ab63801, 1:1000), tubulin (Abcam, Ab3194, 1 μg/ml) and Williams Syndrome Transcription Factor (WSTF; Cell Signaling, 0.3 μg/ml). For IP: NuMA (Oncogene, clone Ab-2 or Bethyl Laboratories) and SNF2h (Abcam, clone 3.25(2)).

### Immunofluorescence

This procedure was performed as described previously (19). Where indicated, immunostaining was preceded with *in situ* cell fractionation to reveal insoluble proteins (24). Fluorescent signals were imaged with a Zeiss CLSM710 using a 63× oil (NA = 1.4) objective. Repair foci were quantified using an automated routine in ImageJ (http://rsbweb.nih.gov/ij/) or by visual scoring. Antibodies used for immunostaining were: 53BP1 (Abcam, ab36823, 5 μg/ml), BRCA1 (Calbiochem, MS110, 0.3 μg/ml), cyclin B1 (Cell Signaling Technology, clone D5C10, 1:200), FLAG (Sigma, M2, 2 μg/ml), γH2AX (Milipore, JBW301, 3.3 μg/ml), Histone H4 acetylated (Milipore, 06–598, 5 μg/ml), Histone H4K20me (Abcam, ab9051, 5 μg/ml), Ki67 (Vector Laboratories, VP-K451, 1:1000), lamin B (Abcam, Ab16048, 2 μg/ml), NuMA (B1C11, 1:2), NuMA full-length (Abcam, ab36999, 1:100), NuMA proximal coiled-coil (Bethyl Laboratories, A301–510A, 4 μg/ml), NuMA distal C-terminal domain (Abcam, clone EP3976, 1:250), RAD51 (Abcam, ab63801, 1:1000), SNF2h (Abcam, ab3749, 15 μg/ml or clone 3.25(2), 4 μg/ml) and WSTF (Abcam, clone EP1704Y, 1:100). Note that for immunostaining with RAD51 antibodies, a permeabilization step with 0.5% triton X100 was performed after fixation with paraformaldehyde.

### Expression vectors and small interfering RNA

Lipofectamine 2000 (Invitrogen) was used to transfect plasmid DNA and siRNA (50 nM; ON-TARGETplus, Dharmacon) in S1, MCF-7 and U2OS cells. For MCF-7 HR-GFP cells, the Fugene (Roche) and HiPerFect (Qiagen) reagents were used to deliver plasmids and siRNA (10 nM), respectively. K562 cells were transfected by electroporation. Incubation times with siRNAs were 6 days (S1 cells) or 4 days (MCF-7 and U2OS cells). Stable cell lines were selected and maintained with G418 (400 μg/ml). To generate SNF2h fused to the green fluorescent protein (SNF2h-GFP), the SNF2h coding sequence was amplified by polymerase chain reaction (PCR) from a cDNA clone (Origene; accession # NM_003601.2) and cloned in frame with GFP in a modified pcDNA3.1 vector. GFP was substituted with mCherry to produce SNF2h-mCherry. Expression vectors for GFP-53BP1 (53BP1's tudor domain fused to GFP; (25)), GFP-CtIP (26), GFP-NuMA (27), GFP- and RFP-PCNA (28), SMRAD51 (29), and SNF2h_1–643_-GFP and GFP-SNF2h_644–1053_ (6) have been described previously. The WSTF cDNA clone including a FLAG epitope tag was a gift from Dr Varga Weisz (The Babraham Institute, UK).

### Fluorescence correlation spectroscopy (FCS)

FCS traces were collected in live cells expressing GFP-tagged proteins. For each cell, triplicate measurements were recorded in the nucleus and the corresponding diffusion times were averaged. FCS measurements were performed on a customized system comprised of an Olympus IX71 microscope and a scanning confocal module (Microtime 200, PicoQuant GmbH) for time-correlated single photon counting time-tagged time-resolved measurements (Time Harp 200, PicoQuant GmbH). A picosecond pulsed 467 nm laser line was used as excitation source for GFP via a water immersion objective (60×, NA = 1.2). Emitted fluorescence was collected using the same objective and filtered from the excitation light by a dichroic mirror (z467/638rpc, Chroma). Fluorescence signals were selected through a 50 μm pinhole to exclude the background noise and out-of-focus fluorescence, and finally recorded by a single photon avalanche photodiodes (SPCM-AQR-14, PerkinElmer Inc.) after passing the 520–40 (Chroma) band pass filter. Collected fluorescence fluctuation was autocorrelated using the software SymPhoTime (PicoQuant GmbH) and fitted with a least-square algorithm.

### Fluorescence lifetime imaging microscopy

Fixed cells were stained with SNF2h (3.25(2)) and NuMA (EP3976) antibodies labeled with Alexa Fluor^®^ 488 and -555 dyes, respectively. Fluorescence lifetime measurements were performed on the custom system described above. Time-correlated single photon counting decay curves were fitted by double exponential using the SymphoTime software (PicoQuant) to obtain fluorescence lifetimes. Fluorescence resonance energy transfer (FRET) efficiencies (*E*) were calculated using the equation: *E* = 1−(tDA/tD), with *τ*DA and *τ*D the donor excited state lifetime in the presence and absence of acceptor, respectively.

### Laser microirradiation

U2OS cells cultured in 35 mm coverglass dishes were treated with 10 μM BrdU for 48 h. The 405 nm laser from a Zeiss CLSM710 microscope was used at maximum power to scan lines across nuclei (100 μs dwelling time; ∼1 s scan/line) to induce DNA damage. A 63× water immersion objective (NA = 1.2) was used for irradiation and imaging. Cells were maintained at 37°C and 5% CO_2_ throughout the experiment. The position of the laser line used to induce DNA damage was recorded and used as peak position to generate the intensity plots. For S1 cells, BrdU was omitted and laser microirradiation time was either ∼1 s or ∼10 s scan/line. Cells were incubated 30 min at 37°C, fixed with paraformaldehyde and processed for immunostaining.

### Repair assays and cell cycle analysis

HR was assessed in MCF-7 and K562 cells with chromosomally integrated or transiently transfected GFP reporters (HR-GFP) (30). Within each biological replicate, values were normalized to the averaged value of the control. NHEJ was measured using a PCR-based assay as described (22). Briefly, genomic PCR with primers flanking the I-SceI site within the chromosomally integrated HR-GFP reporter was performed to measure religation after I-SceI cleavage. Band intensities were normalized to PCR amplification of *GAPDH*. Cell cycle determination of propidium iodide-stained cells was performed on a Beckman Coulter FC500 or a BD Biosciences FACSCalibur flow cytometer as described (31).

### Chromatin texture descriptors

Confocal images of DAPI-stained nuclei were obtained for each condition. These images were converted to 8-bit grayscale images and enhanced by optimizing brightness and contrast. To create nuclear regions of interest, images were subjected to Gaussian filtering, fluorescence intensity-based thresholding and contrast enhancement. Binarized masks were then created for each individual nucleus. A set of 104 texture descriptors was extracted from the images as described (32), processed using principal component analysis, dimensionally reduced linearly into three dimensions, and plotted on a graph. These dimensions (referred to as principal components) are orthogonal to each other, and therefore account for most variance in the binary dataset. Each condition was compared to all other conditions using linear determinant analysis, in which pseudoexperiments (*n* = 50) were simulated for each comparison, yielding sensitivity, specificity and accuracy values. High sensitivity, specificity and accuracy values indicate optimal classification. Datasets are considered different when sensitivity and specificity are above 50.

### Statistical analysis

Statistical analysis was performed using Prism (GraphPad Software Inc.). Student's *t* test and one-way analysis of variance (ANOVA) followed by Bonferroni's post hoc test are indicated with the corresponding *P* values in the figures. A *P* value < 0.05 was considered significant.

## RESULTS

### NuMA interacts with the ISWI ATPase SNF2h and controls SNF2h accumulation at DNA damage sites

ATP-dependent chromatin remodelers catalyze rapid changes in the chromatin landscape in response to DNA damage (4). Stimulated by our observation that NuMA organizes chromatin in the context of mammary epithelial cell differentiation (23), we initially examined a potential relation between NuMA and ATP-dependent remodelers by cosedimentation analysis. The sedimentation pattern of NuMA overlapped with that of the ISWI family ATPase SNF2h but not the SWI/SNF family BRG1 ATPase (Supplementary Figure S1a), prompting us to further investigate a possible link between NuMA and SNF2h.

**Figure 1.**
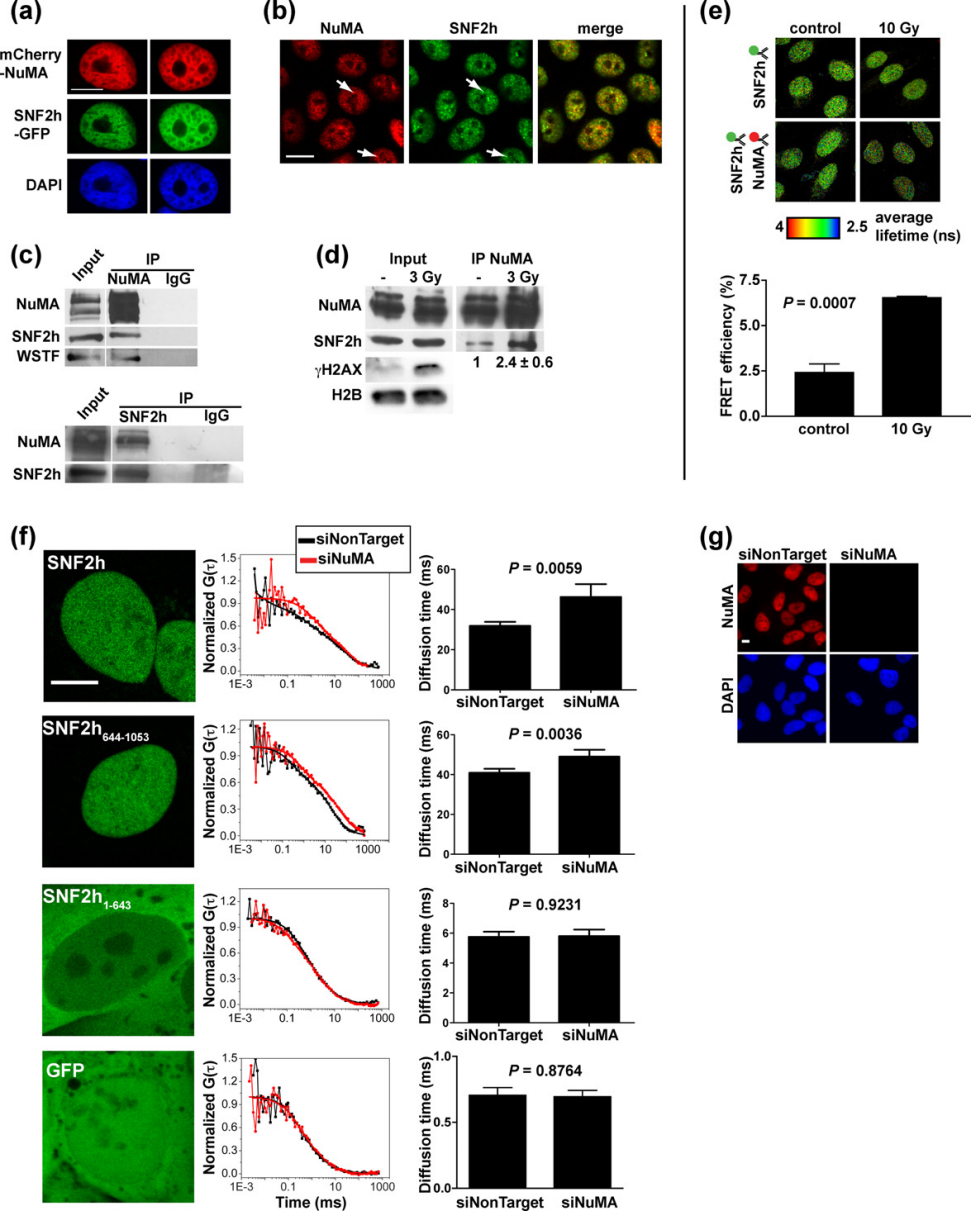
NuMA interacts with SNF2h. (**a**) Confocal images of SNF2h-GFP and mCherry-NuMA transiently coexpressed in non-neoplastic HMT-3522 S1 cells. The nuclei were counterstained with DAPI. (**b**) Colocalization of NuMA and SNF2h analyzed by immunostaining in S1 cells. Arrows indicate colocalized foci. (**c**) IP of NuMA and SNF2h from S1 nuclear extracts. Nonspecific immunoglobulins (IgGs) were used as controls. The western blots were probed for NuMA, SNF2h and WSTF. (**d**) NuMA IP from nuclear extracts of malignant HMT-3522 T4-2 cells either mock irradiated or exposed to 3 Gy of gamma radiations and left to recover for 30 min. SNF2h signal intensity in IP samples was quantified by densitometry and averaged (*n* = 4, bottom of IP panel). DNA damage induction was verified by probing the input samples for γH2AX; H2B was used as loading control. (**e**) FRET measured in nonirradiated (control) and irradiated (10 Gy) U2OS cells using fluorescence lifetime imaging. SNF2h was stained with antibodies coupled to Alexa Fluor^®^ 488 (FRET donor) and NuMA with Alexa Fluor^®^ 555-conjugated antibodies (FRET acceptor). Donor fluorescence lifetime was measured in the absence or presence of acceptor and used to calculate FRET efficiencies. Representative fluorescence lifetime images of the donor are shown. The bar graph represents means ± SEM FRET efficiencies (*n* = 3 experiments; ≥ 25 cells/conditions in each experiment). (**f**) Diffusion of GFP-tagged SNF2h measured by fluorescence correlation spectroscopy in U2OS cells. SNF2h fragments (C-terminal a.a. 644–1053 and N-terminal a.a. 1–643) and GFP alone are used as controls. Cells transfected with NuMA-targeting siRNA (siNuMA) are compared to cells transfected with nontargeting siRNAs (siNonTarget). Representative confocal images (left) and FCS curves (middle) are shown. Diffusion times are represented in the graphs as mean ± SEM (*n* ≥ 20 cells from at least two biological replicates). (**g**) NuMA silencing verified by immunostaining. Scale bars, 10 μm.

Coexpression of NuMA and SNF2h with fluorescent protein tags (Figure 1a) and dual labelling of NuMA and SNF2h with antibodies (Figure 1b) both indicated partial colocalization in the nucleus. In non-neoplastic and cancer cells, NuMA antibodies coprecipitated SNF2h and reciprocally, SNF2h antibodies coprecipitated NuMA. Antibodies against NuMA also coprecipitated WSTF, a SNF2h partner in the WICH [SNF2h-WSTF] complex involved in DNA repair (33,34) (Figure 1c and Supplementary Figure S1b). In contrast, BRG1 was not detected in NuMA IP fractions (Supplementary Figure S1c), consistent with the distinct sedimentation of the two proteins. In cells exposed to gamma irradiation, SNF2h pull-down by NuMA antibodies increased by 2-fold (Figure 1d). FRET between labelled SNF2h and NuMA antibodies confirmed an interaction that increased in response to IR (Figure 1e). These results indicate that NuMA and SNF2h interact under physiological conditions and hint at functional relevance of this interaction in DNA repair.

Considering that NuMA is a large nuclear structural protein, we reasoned that binding to NuMA may alter SNF2h kinetics in the nucleus. Cells with NuMA silenced by siRNA (siNuMA) displayed significantly decreased diffusion of SNF2h-GFP in the nucleoplasm compared to control, as shown with fluorescence correlation spectroscopy (Figure 1f and g). The same effect was measured in cells expressing the C-terminal substrate recognition domains of SNF2h (SNF2h_644–1053_). In contrast, NuMA knock-down had no measurable influence on the diffusion of the N-terminal portion of SNF2h spanning the ATPase domain (SNF2h_1–643_) or on GFP alone. Thus, NuMA specifically controls SNF2h kinetics in the nucleus.

**Figure 2.**
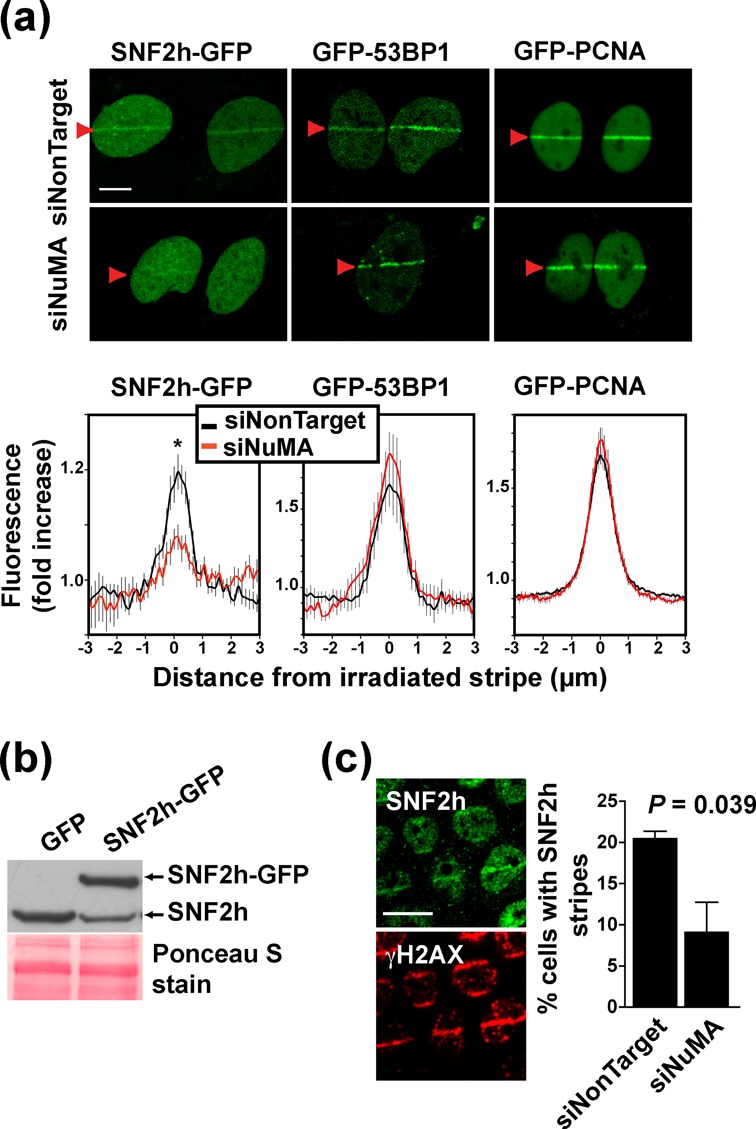
Accumulation of SNF2h at DNA breaks requires NuMA. (**a**) Accumulation of SNF2h-GFP, GFP-53BP1 and GFP-PCNA at laser-microirradiated stripes (arrowheads) in U2OS cells transfected with NuMA siRNA and nontargeting siRNA. Averaged normalized pixel intensities at the irradiation stripes are shown in the bottom panels (*n* ≥ 10 cells; *P < 0.05, unpaired t-test). (**b**) Expression of endogenous and recombinant SNF2h quantified by western blot in SNF2h-GFP or GFP U2OS cells. Equal loading is shown by Ponceau stain. (**c**) Immunostaining for SNF2h and γH2AX in S1 cells. Cells were microirradiated and left to recover for 30 min. The percentages of nuclei with overlapping SNF2h and γH2AX stripes in siRNA-transfected cells are presented as mean ± SEM in the bar graph (*n* = 3 biological replicates with > 50 cells/condition in each experiment). Scale bars, 10 μm.

The next logical step was to verify whether NuMA also impacts on SNF2h recruitment to DSBs. Using laser microirradiation, we confirmed previous knowledge that full-length SNF2h and the C-terminal portion of the protein (SNF2h_644–1053_)—but not the N-terminal SNF2h fragment (SNF2h_1–643_)—rapidly and persistently accumulate at damage sites (Supplementary Figure S2a) (6). In cells silencing NuMA, the percentage of cells accumulating SNF2h-GFP at irradiation stripes (and the intensity of SNF2h-GFP at the stripes) was significantly lower compared to controls (54.8 ± 6.0% and 83.7 ± 4.6%, respectively; *P =* 0.019, Figure 2a and b. Recruitment of the repair factors 53BP1 and proliferating cell nuclear antigen (PCNA) was not affected by NuMA silencing, ruling out pleiotropic effects of NuMA disruption on the DDR (Figure 2a). Accumulation of endogenous SNF2h after laser microirradiation was also reduced in cells silencing NuMA (Figure 2c).

To determine if NuMA is also necessary for the recruitment of the SNF2h partner WSTF, epitope-tagged WSTF was expressed in U2OS cells. The recombinant protein accumulated at the microirradiation stripes (Supplementary Figure S2b) but proved to be cytotoxic. Instead, we relied on immunostaining of endogenous WSTF. Similar percentages of cells with WSTF stripes were calculated in siNuMA and control transfections. Moreover, WSTF accumulated at microirradiation stripes even in cells with no detectable NuMA (Supplementary Figure S2c). We conclude that for the WICH complex, the dependency on NuMA for accumulation at DNA damage sites is specific to SNF2h.

### NuMA accumulates at sites of DNA damage

**Figure 3.**
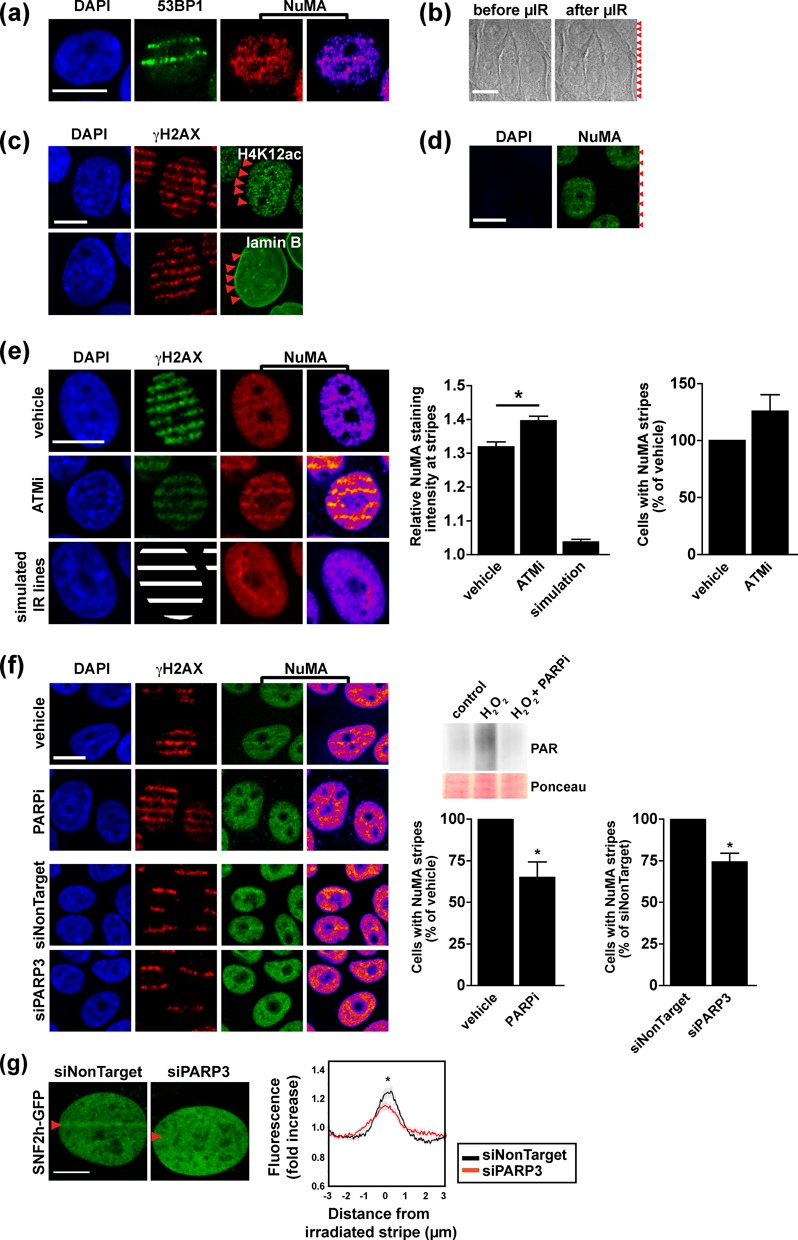
NuMA accumulates at DNA damage sites. (**a**) Immunostaining for NuMA and 53BP1 (used as marker of DNA damage) in S1 cells 30 min after laser microirradiation. NuMA signal intensities are visualized with a heat map. (**b**) Bright field images of cells before and after microirradiation (red arrowheads). (**c**) Acetylated histone 4 (H4K12ac), lamin B and γH2AX detected by immunostaining in cells microirradiated as in A. (**d**) NuMA detection after laser microirradiation and *in situ* extraction of soluble and chromatin-bound proteins. Weak DAPI staining confirms release of DNA. (**e**) Dual NuMA-γH2AX immunostaining after laser microirradiation. Cells were treated with vehicle or with the ATM inhibitor KU55933 (ATMi, 10 μM, 2 h). NuMA staining intensity was measured at irradiated regions defined using γH2AX signals. In nonirradiated cells, simulation stripes were drawn as surrogate for γH2AX. Pixel intensities at microirradiation and simulation stripes are normalized to the average intensity in the nucleus (left graph; *n* ≥ 80 cells/condition; *P < 0.001, ANOVA and Tukey's multiple comparison test). The relative fraction of cells with NuMA stripes is also shown (right graph; *n* = 4 biological replicates). (**f**) Analysis as in (e) of cells treated with the PARP inhibitor IQD (PARPi, 30 μM, 2 h) or transfected either with nontargeting siRNA or with PARP3 siRNA (*n* ≥ 3 biological replicates; *P < 0.05, one sample t-test). PARP inhibition was validated by western blot in S1 cells treated with H_2_O_2_ (10 mM, 10 min) in the presence or absence of PARPi (inset). Ponceau stain is shown as loading control. (**g**) SNF2h-GFP accumulation at laser-microirradiated stripes in U2OS cells transfected with PARP3 siRNA and nontargeting siRNA. Averaged normalized pixel intensities at the irradiation stripes are shown in the graph (*n* ≥ 15 cells; *P < 0.05, unpaired t-test). Scale bars, 10 μm.

Factors regulating SNF2h accumulation at DNA breaks are likely to be themselves responsive to DNA damage. To explore NuMA's behavior specifically at DNA damage sites, laser-microirradiated cells were immunostained for NuMA. A subtle but significant accumulation of NuMA was measured at the microirradiation stripes using three different NuMA antibodies (Figure 3). Laser microirradiation did not alter cell morphology, nor did it induce apoptosis as shown by DAPI stain, and the majority of DNA lesions triggered by the laser were repaired within 24 h (Figure 3b and Supplementary Figure S3a). Moreover, histones and lamin B did not aggregate at DNA damage sites under the conditions used for microirradiation (Figure 3c). These observations suggest *bona fide* accumulation of NuMA at DNA breaks and that this phenomenon presages DNA repair rather than cell death.

NuMA is a component of the nucleoskeleton (19), a dynamic structure that resists extraction with high salt and DNAse digestion. NuMA is also associated with the chromatin compartment (23). We reasoned that if NuMA served as a platform regulating SNF2h in the DDR, this function might rely on its nucleoskeletal fraction. As expected, nuclei retained intense NuMA signals at microirradiation stripes after *in situ* extraction of soluble and chromatin-bound proteins (Figure 3d).

The phosphatidylinositol 3-kinase (PI3K)-like protein kinase ATM mediates chromatin remodeling (15,35,36). Mass spectrometry analyses of protein phosphorylation in response to DNA damage indicate that NuMA is phosphorylated by ATM in response to UV, IR and etoposide treatments (14–17). We confirmed that at least one site of NuMA (the serine at position 395) is an ATM-specific substrate; phosphorylation was observed within minutes after IR exposure and could be blocked with a specific ATM inhibitor (KU55933, ATMi) or by a null ATM mutation (Supplementary Figure S3b–d). Microirradiated cells treated with ATMi displayed apoptotic nuclear features, persistence of γH2AX, and an increased presence of NuMA at microirradiation stripes, whereas ATMi was nontoxic for cells in nonirradiated regions (Figure 3e and Supplementary Figure S3a). The results indicate that phosphorylation by ATM is not necessary for the accumulation of NuMA at DNA damage sites. SNF2h staining signals at DNA damage sites were also more pronounced upon ATM blockade (Supplementary Figure S3e). Increased NuMA and SNF2h densities at DNA damage stripes in ATMi-treated cells may reflect a higher number of unrepaired lesions caused by ATM inhibition, although we cannot exclude that phosphorylation is necessary for the release of NuMA from repair sites, as it is the case for KAP-1 (37).

NuMA is a known acceptor of poly(ADP-ribose) chains during mitosis (38) and interacts with PARP3, a poly(ADP-ribose) polymerase implicated in the cellular responses to DSB (39). PARP inhibition using 1,5-Isoquinolinediol (IQD) as well as silencing of PARP3 decreased the presence of NuMA at laser microirradiation stripes (Figure 3f). Importantly, PARP3 knock-down also significantly reduced the accumulation of SNF2h-GFP at laser microirradiated sites (Figure 3g).

We conclude that the enrichment of NuMA at DNA damage sites involves an insoluble, nucleoskeletal fraction of the protein and that accumulation of both NuMA and SNF2h at DNA breaks depends on PARP activity rather than phosphorylation by ATM.

### NuMA is necessary for the efficient recruitment of repair factors and DSB repair

**Figure 4.**
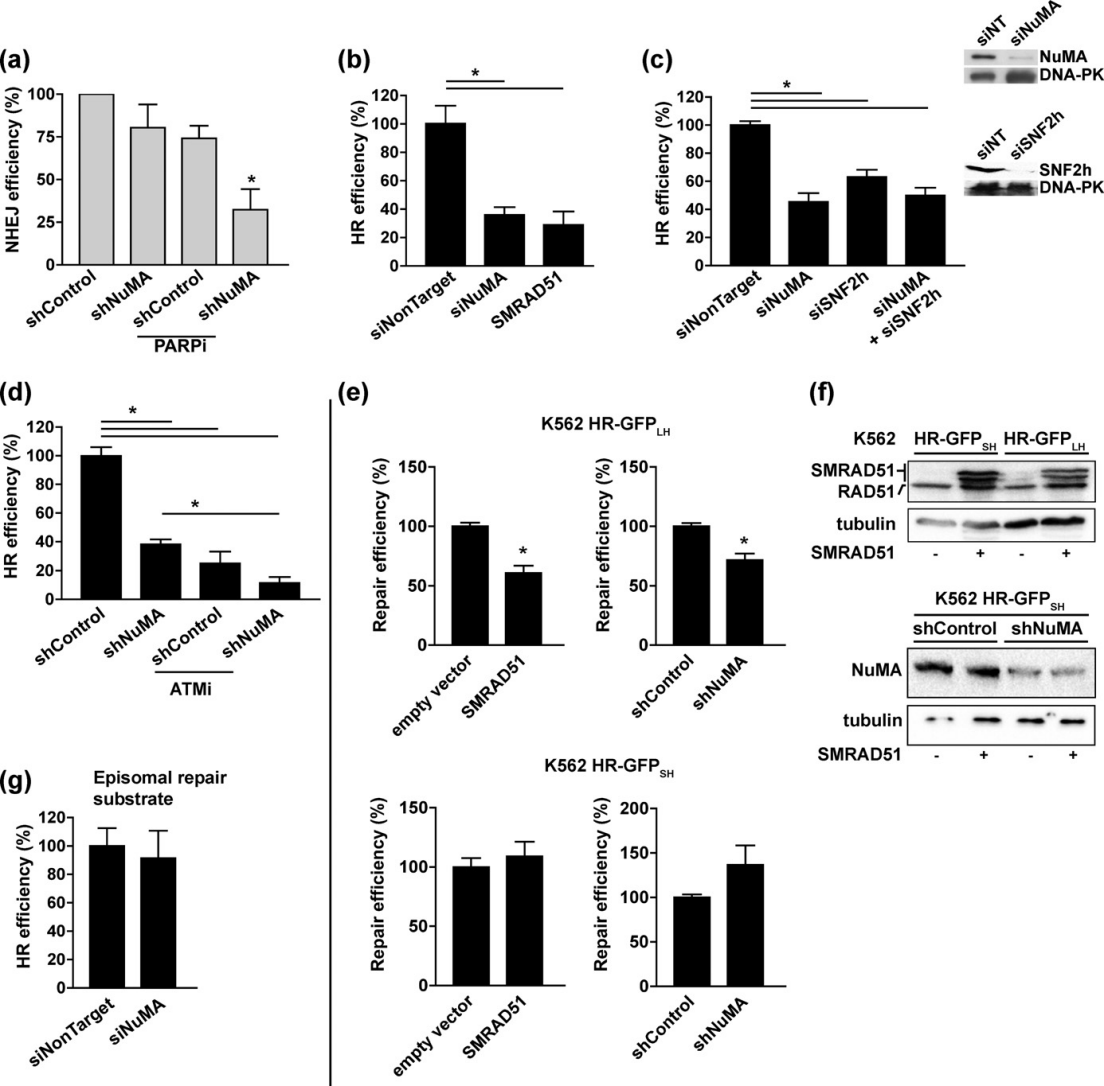
NuMA is required for efficient repair of DSBs. (**a**) Canonical NHEJ after I-SceI-induced DSBs in MCF-7 cells with a genomic integration of a repair substrate containing a meganuclease recognition site. Cells were transfected with shRNA constructs specific for NuMA or nontargeting (shControl), and reconstitution of the I-SceI site by end-joining was quantified using genomic PCR. Where indicated, cells were treated with IQD (PARPi, 200 μM, 48 h; *n* = 3; *P < 0.05, one sample t-test). (**b-d**) HR repair measured in MCF-7 cells with a stable genomic integration of the HR-GFP reporter. The frequency of GFP-positive events was quantified by flow cytometry (*n* = 3–5 experiments with measurements in triplicates, each corrected by individual transfection efficiency; ^*P^ < 0.05, ANOVA and Tukey's multiple comparison test). (**b**) Cells were transfected with NuMA siRNA or with nontargeting siRNA and with the cDNA of a dominant-negative form of RAD51 (SMRAD51), as well as with I-SceI for DSB induction. (**c**) Cells were transfected with NuMA and/or SNF2h siRNA as indicated. NuMA and SNF2h silencing was verified by western blot, using DNA-PK as loading control (inset). (**d**) Cells were transfected with shRNA plasmids and treated with KU55933 (ATMi, 10 μM, 48 h) as indicated. (**e**) Homology-dependent DNA repair in K562 cells with a stable integration of a HR-GFP reporter with long homologies (HR-GFP_LH_; top) or with short homologies (HR-GFP_SH_; bottom). Cells were transfected with SMRAD51 and with shRNA as indicated. I-SceI-mediated cleavage is repaired in part by RAD51-dependent HR in HR-GFP_LH_ cells but not in HR-GFP_SH_ cells, as illustrated by SMRAD51 sensitivity. (**f**) Western blot showing expression of SMRAD51 and depletion of NuMA in cells treated as in E; tubulin was used as loading control. (**g**) HR repair of episomal DNA in MCF-7 cells after transient transfection of the HR-GFP plasmid.

The possibility that NuMA impacts DNA repair via its influence on SNF2h was first assessed by measuring canonical NHEJ activity by PCR in MCF-7 cells with a genomically integrated I-SceI meganuclease site (22). NuMA silencing did not significantly alter NHEJ activity after I-SceI cleavage. However, combining NuMA silencing and PARP inhibition with IQD synergistically reduced NHEJ activity (Figure 4a). This effect may be explained by the inhibition of two independent NHEJ events, conceivably PAR3-mediated retention of XRCC4 and DNA ligase IV (40), and chromatin remodeling by SNF2h.

Then, we used a GFP-based assay system (HR-GFP) in which homologous DSB repair reconstitutes a functional GFP gene after DSB induction with I-SceI (30). In MCF-7 cells with a stable genomic integration of HR-GFP, NuMA silencing using si- or shRNA led to a 60% decrease in HR (Figure 4b–d), an effect comparable to the expression of a dominant-negative form of RAD51 (29) (Figure 4b). HR has been shown to rely on SNF2h function (6,7,41–43), and we confirmed this observation in MCF-7 HR-GFP cells after SNF2h knock-down (Figure 4c). Noticeably, silencing SNF2h reduced HR to a similar extent compared to NuMA silencing, and simultaneous knock-down of SNF2h and NuMA did not lead to additive effects, indicating epistasis between the two genes. In contrast, blocking ATM in cells silencing NuMA further decreased HR activity, suggesting that ATM regulates HR independently from NuMA (Figure 4d). HR activity also decreased upon NuMA silencing in erythroleukemic K562 cells and correlated with repair sensitivity to SMRAD51 expression (Figure 4e and f. These results indicate that NuMA is implicated in RAD51-dependent HR. Importantly, there was no measurable defect of extrachromosomal DNA repair in cells transiently transfected with HR-GFP (Figure 4g). Hence, the role of NuMA in HR seems restricted to the chromatin context. It is unlikely that the effects of NuMA on HR were due to cell cycle imbalance since only a modest influence of NuMA silencing on cell cycle distribution was measured in MCF-7 cells and NuMA knock-down did not affect the cell cycle in K562 cells (Supplementary Figure S4a).

**Figure 5.**
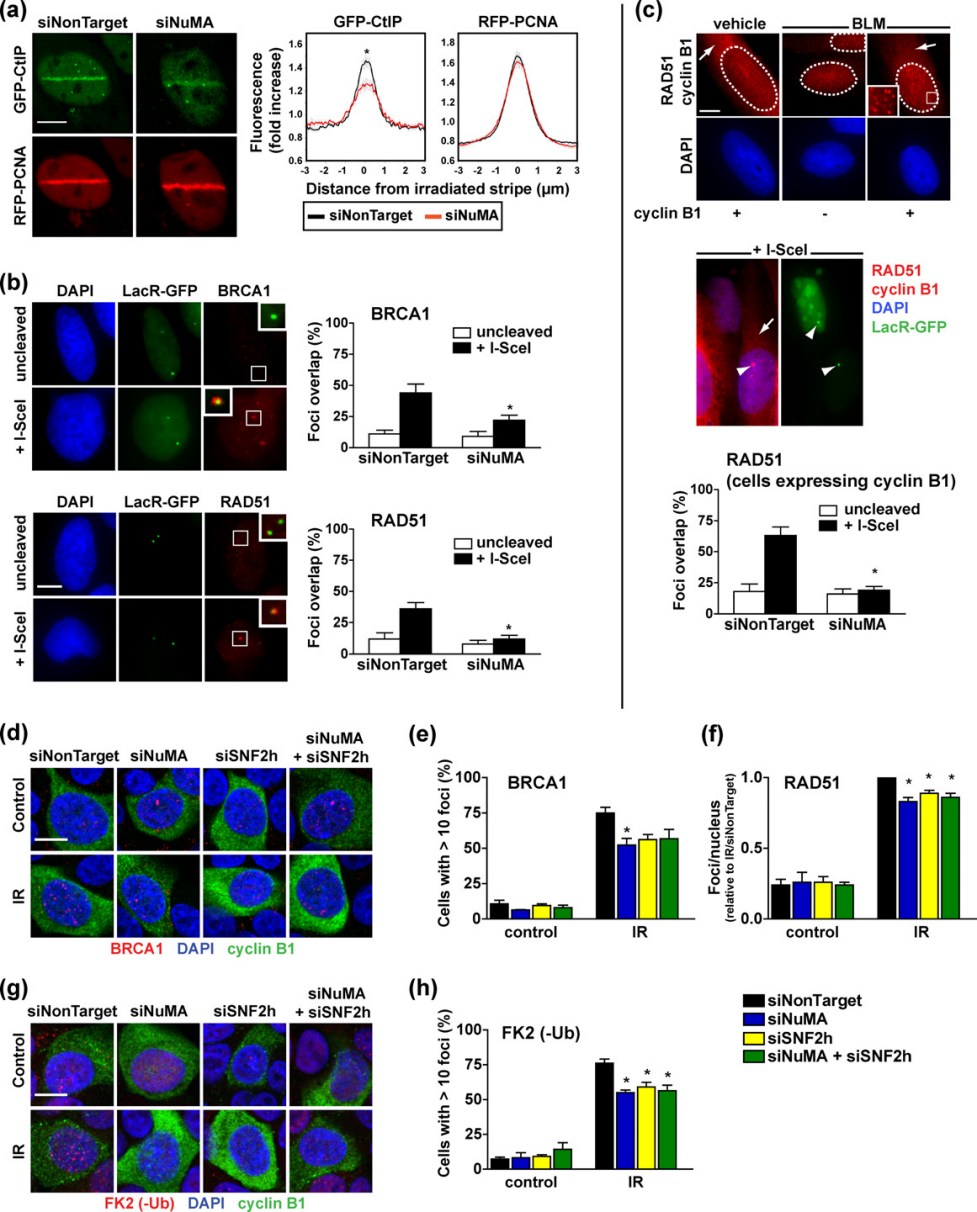
NuMA modulates the recruitment of HR factors at DNA breaks. (**a**) Accumulation of GFP-CtIP and RFP-PCNA at laser-microirradiated stripes in U2OS cells transfected with either NuMA siRNA or nontargeting siRNA. Averaged normalized pixel intensities at the irradiation stripes are shown on the right (*n* = 20 cells; *P < 0.05, unpaired t-test). (**b**) BRCA1 and RAD51 foci formation at I-SceI cleavage sites. U2OS cells carrying a stable genomic integration of the I-SceI recognition site flanked by LacO repeats were transfected with I-SceI (+I-SceI) to induce DSBs, LacR-GFP to visualize DSB positions and siRNA as indicated. I-SceI cDNA was omitted in uncleaved controls. Overlap between LacR-GFP and RAD51 or BRCA1 immunostaining signals was quantified and is shown in the bar graphs (*n* = 4 biological replicates; *P < 0.05, unpaired t-test compared to siNonTarget). (**c**) Quantification of RAD51 foci as in B, guided by cyclin B1 staining (*n* = 3; *P < 0.005, unpaired t-test). Cytoplasmic cyclin B1 signals (arrows) were used to discriminate HR-competent late S/G2 phase from the G1/early S phase populations in U2OS cells. In the same fluorescence channel, RAD51 staining (nuclear signals) labeled DNA repair foci. RAD51 foci formation in bleomycin (BLM)-treated cells and at cleaved I-SceI arrays (LacR-GFP foci; arrowheads) was only observed in cyclin B1-positive cells. (**d**) Immunostaining of BRCA1 foci in S1 cells transfected with NuMA-specific, SNF2h specific or nontargeting siRNA. Cells were irradiated (IR, 3 Gy) and left to recover for 3 h. Confocal images with merged BRCA1 (red), cyclin B1 (green) and DAPI (blue) signals are shown. (**e**) Quantification of BRCA1 foci in cells treated as in D. Cyclin B1 signals were used to select HR-competent cells. Results represent mean ± SEM (*n* = 3, *P < 0.05, ANOVA and Tukey's post-hoc test, compared to siNonTarget). (**f**) RAD51 foci formation in S1 cells treated as in D (*n* = 3; *P < 0.05, one sample t-test relative to siNonTarget). (**g**) Confocal images and (**h**) quantification of conjugated ubiquitin (-Ub) foci detected with the FK2 antibody in cells treated as in D (*n* = 3, *P < 0.05, ANOVA and Tukey's). Scale bars, 10 μm.

A committing step of RAD51-dependent HR repair is the initiation of DSB end resection by CtIP (26). Since NuMA depletion compromises HR, we assessed whether the loss of NuMA affects the response of CtIP to DNA damage. Silencing NuMA significantly reduced the fluorescence amplitude of GFP-CtIP (but not of coexpressed RFP-PCNA) at laser-microirradiated stripes (Figure 5a). DNA resection enables the sequential recruitment of HR factors to DNA breaks. To examine the role of NuMA in HR factor recruitment, we used U2OS cells with an I-SceI site flanking LacO arrays (18,44). In this system, nuclear expression of I-SceI generates DSBs that can be localized with GFP-tagged Lac repressors (LacR-GFP). Accumulation of BRCA1 and RAD51 at cleaved LacO arrays was significantly reduced in cells transfected with NuMA siRNA compared to nontargeting siRNA (Figure 5b). In order to control for the effect of NuMA on cell proliferation (Supplementary Figure S4a), the analysis of RAD51 foci was guided using cyclin B1, a marker of late S/G2 phases. As expected, only cells with cytosolic cyclin B1 signals displayed RAD51 repair foci in response to DSB induction with bleomycin, and overlap between LacR-GFP signals and RAD51 staining at cleaved arrays was limited to cells with cyclin B1 expression, indicating that HR-competent cells were effectively selected (Figure 5c). Using cyclin B1-guided analysis, decreased RAD51 accumulation at DSBs was again measured in cells silencing NuMA, indicating that the effect of NuMA on HR does not merely reflect changes in cell proliferation. We ruled out the possibility that NuMA silencing altered the expression of BRCA1 and RAD51, as well as SNF2h and 53BP1—an important protein in repair pathway choice (45,46) (Supplementary Figure S4).

To firmly establish the role of NuMA in HR, we quantified the HR response after irradiation of S1 cells. Silencing NuMA reduced the formation of BRCA1 and RAD51 foci in HR-competent S1 cells (Figure 5d–f), but it did not alter proliferation (Supplementary Figure S4a and b). Mirroring the observations with the HR-GFP system, the accumulation of BRCA1 and RAD51 foci in irradiated S1 cells was similarly impaired by silencing of NuMA, SNF2h or both genes, which confirms that NuMA and SNF2h function through a common pathway.

SNF2h interacts with the E3 ubiquitin ligase RNF168 and mediates RNF168 accumulation and function at DSBs (42). In turn, histone polyubiquitination by RNF168 enables the assembly of the BRCA1-A complex which initiates HR (47). In S1 cells, the FK2 antibody detected heterogeneous focal accumulation of conjugated ubiquitin, likely reflecting the multiple roles of ubiquitin conjugates in DNA repair, transcription regulation and histone turnover (48). Despite heterogeneity, a significant increase of conjugated ubiquitin foci was measured after irradiation in HR-competent cells and, as expected, silencing SNF2h reduced this effect (42); (Figure 5g and h. Consistent with the scenario of a NuMA-dependent SNF2h function, silencing NuMA and dual silencing of NuMA and SNF2h led to similar decreases in conjugated ubiquitin foci.

**Figure 6.**
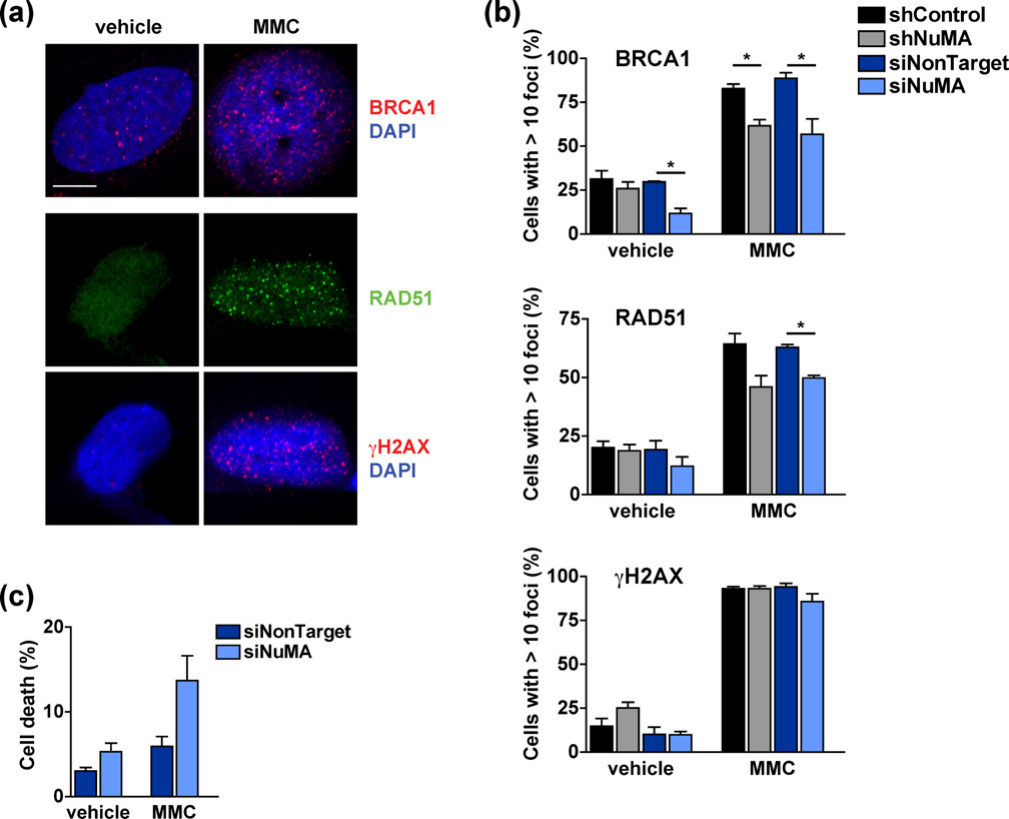
NuMA mediates DNA repair and cell survival in cells exposed to MMC. (**a-b**) Formation of BRCA1, RAD51 and γH2AX repair foci in U2OS cells treated with MMC (18 h, 2.6 μM) or vehicle. Shown are representative confocal images (a) and foci quantification in cells transfected with NuMA and nontargeting si- or shRNA (b) (*n* = 3, *P < 0.05, unpaired t-tests). (**c**) Cell viability determined by trypan blue staining in cells treated as in (b) (*n* = 2). Scale bar, 10 μm.

The repair of interstrand cross-links generated by the chemotherapeutic drug mitomycin C (MMC) relies on HR (49). Decreased BRCA1 and RAD51 foci formation was measured in MMC-treated cells silencing NuMA compared to controls, ascertaining the involvement of NuMA in HR factor assembly (Figure 6a and b. The fraction of cells with γH2AX signals was similar across transfection conditions, denoting that silencing NuMA did not reduce MMC-induced DSB formation. Moreover, increased MMC-mediated cell death was measured in cells with NuMA knocked-down, demonstrating that the differences in HR foci formation reflected defective repair rather than decreased MMC effectiveness upon NuMA silencing (Figure 6c).

We conclude from this series of experiments that NuMA is required for IR-induced formation of ubiquitin conjugates, timely recruitment of CtIP and BRCA1, as well as RAD51 filament formation and thus, homology-directed DNA repair. This function of NuMA appears to be mediated by SNF2h and does not result from NuMA's influence on the cell cycle.

### NuMA maintains H2AX phosphorylation and influences chromatin reorganization after DNA damage

**Figure 7.**
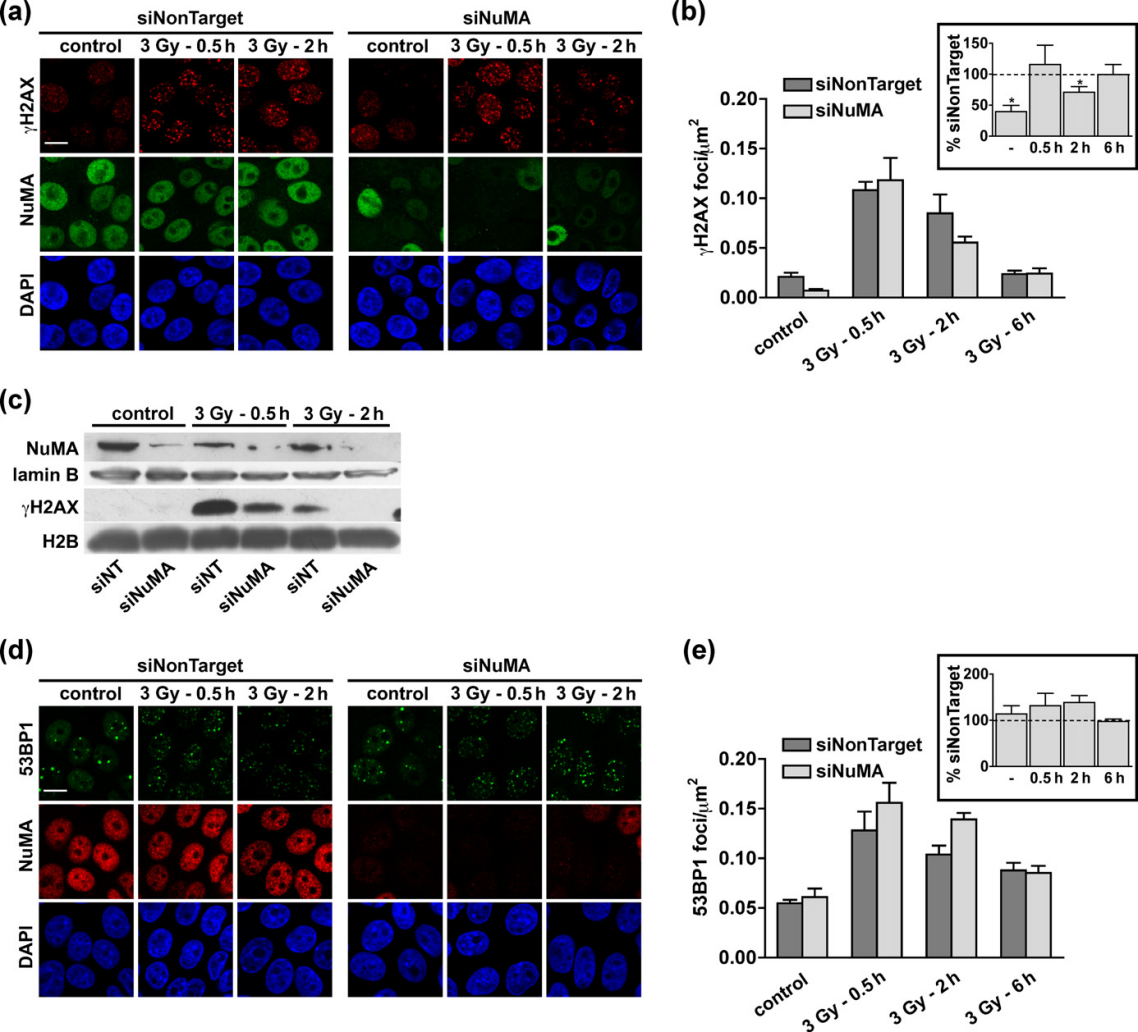
Maintenance of γH2AX foci requires NuMA. (**a–b**) Accumulation of γH2AX in S1 cells transfected with NuMA-specific or with nontargeting siRNA. The cells were irradiated (3 Gy) and left to recover for 30 min, 2 h and 6 h before immunostaining for γH2AX and NuMA. (**a**) Representative confocal images. (**b**) Quantification of foci densities. Cells treated with NuMA siRNA that retained NuMA expression were excluded from the analysis. Values normalized to the nontarget siRNA transfection are shown in the inset (*n* = 4; *P < 0.05, one-sample t-test). (**c**) Western blot analysis using antibodies against γH2AX, NuMA (to assess silencing), as well as lamin B and H2B (loading controls) of S1 cells treated as in A–B. (**d**) Confocal images and (**e**) quantification of 53BP1 foci formation in S1 cells treated as in (a–b) (*n* = 4). Scale bars, 10 μm.

Phosphorylation of H2AX by PI3K-like kinases is an essential histone modification in the DDR; it occurs within minutes of DSB formation. We previously measured reduced levels of γH2AX after persistent DNA damage induction with the radiomimetic drug bleomycin and with I-SceI in cells silencing NuMA (18), which could reflect either a reduced amplitude of the γH2AX response or a failure to maintain this mark. To distinguish between these two possibilities, we quantified γH2AX foci over time after IR exposure. The peak foci density 30 min after IR was indistinguishable between siNuMA and control cells (Figure 7a and b; Supplementary Figure S5a and b). A higher IR dose (10 Gy) yielded denser γH2AX foci, ruling out saturation of foci counting (Supplementary Figure S5c). These results indicate that NuMA silencing does not alter the initial DDR by PI3K-like protein kinases. In contrast, we measured a 29% reduction (*P* = 0.047) of γH2AX foci density in siNuMA-transfected S1 cells compared to controls 2 h after IR exposure (Figure 7a and b. MCF-7 cells displayed a similar response (Supplementary Figure S5a and b), and western blot analysis confirmed that H2AX phosphorylation was not retained upon siNuMA transfection in both cell lines (Figure 7c and Supplementary Figure S5d). The γH2AX response in mammary epithelial cells is influenced by the cell cycle status (50). Here, it is unlikely that the effect of NuMA silencing on γH2AX merely reflects cell cycle imbalance. As presented in the previous paragraph, NuMA silencing in S1 cells did not alter cell cycle distribution. Moreover, premature loss of γH2AX foci was also measured after silencing NuMA in serum-starved MCF-7 cells with reduced proliferation (Supplementary Figure S5e); this phenomenon occurred irrespective of the Ki67 proliferation marker status (Supplementary Figure S5f). Unlike the γH2AX mark, cells silencing NuMA accumulated slightly more 53BP1 foci compared to control at the two-hour recovery time point (Figure 7d and e, consistent with a repair defect in cells silencing NuMA. Hence, the rapid loss of γH2AX in siNuMA transfected cells does not reflect faster repair, but rather compromised maintenance of the mark.

**Figure 8.**
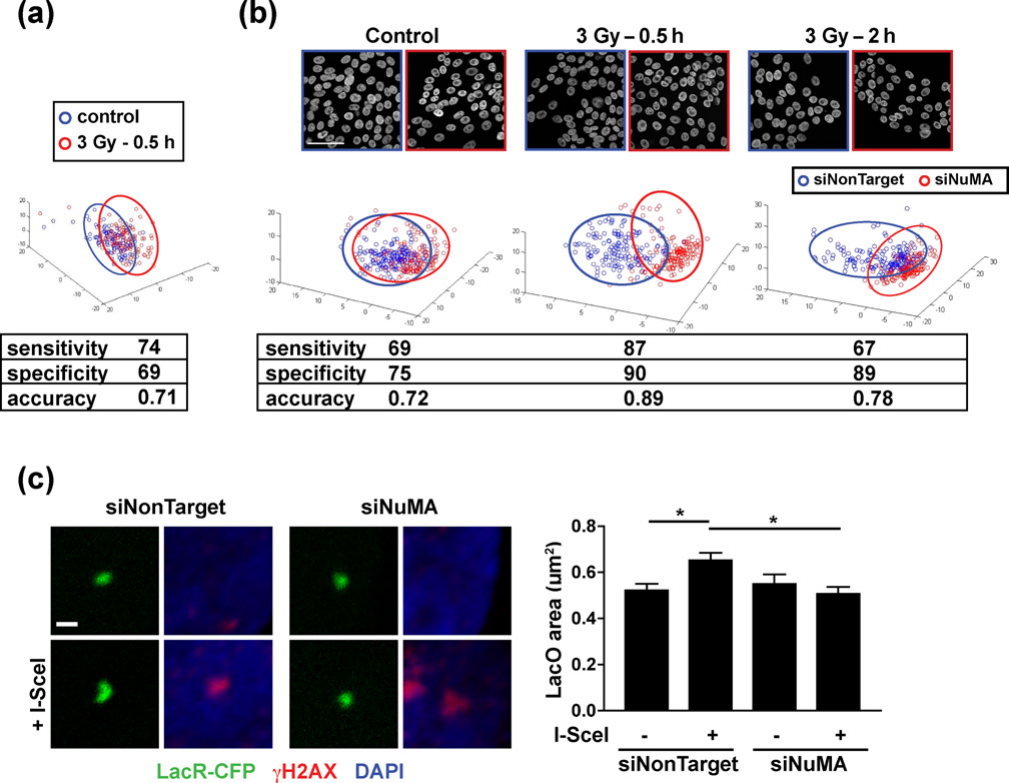
The chromatin response to DNA damage is altered in cells silencing NuMA. (**a**) Texture descriptors extracted from confocal images of DAPI-stained nuclei from mock-irradiated (control) or irradiated (3 Gy) cells. The descriptors were condensed to a three-dimensional composite feature space with principal component analysis. Each point on the graph represents one nucleus. Data are from four biological replicates. High specificity, sensitivity and accuracy values indicate separation of the two cell populations. (**b**) DAPI texture analysis performed as in A in cells transfected with NuMA siRNA or with nontargeting siRNA. Irradiated cells were left to recover for 30 min or 2 h. (**c**) Measurement of cleaved (+I-SceI) and uncleaved LacO array size in U2OS cells treated with NuMA siRNA or with nontargeting siRNA. Images show LacO arrays labeled with LacR-CFP and γH2AX (used to verify presence/absence of cleavage). Values in the graph represent mean ± SEM (*n* ∼ 50 cells from three experiments; *P < 0.05, ANOVA and Tukey's multiple comparison test). Scale bars, 50 μm (b) and 1 μm (c).

The interaction between NuMA and SNF2h and the premature loss of γH2AX in cells silencing NuMA strongly impart that NuMA controls chromatin remodeling during the DDR. In a first approach, we applied high-content morphometric descriptor analysis (32) to DAPI images, using DAPI as a proxy for chromatin organization (Supplementary Figure S5g). This technique permits a precise analysis of cellular phenotypes based on multidimensional image texture measurements. For visualization, the datasets are subjected to principal component analysis, yielding three-dimensional feature spaces with nuclei represented as points. In our experiments, proximity between two points indicates similar DAPI textures. In this assay, nuclei from S1 cells exposed to IR could be distinguished from controls (Figure 8a), consistent with changes in higher-order chromatin structure during the DDR (51). Nuclei from siNuMA and control cells could also be distinguished based on DAPI texture. Importantly, a clear segregation of siNuMA transfectants from controls was measured 30 min after IR, illustrating that NuMA influences chromatin organization after DNA damage. The difference persisted but was attenuated during recovery (Figure 8b), Similar observations were made in MCF-7 cells (Supplementary Figure S5h).

To directly measure the impact of NuMA on chromatin at DNA damage sites, we used the U2OS LacO/I-SceI system in which a defined chromatin region is visualized by expressing LacR fused to a fluorescent protein. We quantified the size of LacR-CFP foci in the absence or presence of I-SceI cleavage. As anticipated, DSB induction with I-SceI elicited a significant expansion of LacR-CFP foci (Figure 8c). This apparent decondensation of the chromatin at DNA break sites was not observed in cells silencing NuMA, confirming a role for NuMA in the chromatin response to DNA damage.

## DISCUSSION

The function of chromatin remodelers must be finely orchestrated to ensure the precise nucleosome reorganization necessary for transcription, DNA synthesis and DNA repair. We report that the structural nuclear protein NuMA controls the activity of the ISWI ATPase SNF2h in DNA repair. The findings support the postulated significance of NuMA expression in the cell nucleus during interphase (19) and substantiate a function for NuMA in chromatin remodeling (23).

SNF2h has been proposed to sample chromatin by transient, genome-wide associations with nucleosomes before being stabilized at specific sites such as damaged DNA and replication forks (5). Our data indicate that NuMA is one of the stabilizing factors since the interaction between NuMA and SNF2h is reinforced during the DDR, NuMA regulates the diffusion of SNF2h, and it is necessary for the accumulation of SNF2h at laser microirradiation sites. Importantly, NuMA itself also accumulates at sites of DNA damage. While the mechanisms regulating NuMA's presence at DNA breaks are not clear yet, our results indicate that NuMA recruitment and/or stabilization at damaged chromatin involves PARylation. We did not detect NuMA PARylation in response to IR or hydrogen peroxide (data not shown) and NuMA was not among the PARP substrates identified during the DDR by a recent proteomics study (52). We cannot exclude that a small fraction of nonextractable NuMA, at or near break sites, becomes PARylated, but we favor the possibility that NuMA interacts on site with PAR chains. Indeed, NuMA binds PARP3 (39) that accumulates at DNA breaks, and the coiled-coil domain of NuMA associates with PAR chains *in vitro.* This association was proposed to favor microtubule clustering at the spindle pole during mitosis (53). We suggest that PAR chains could also promote NuMA's accumulation at damaged chromatin, leading to the stabilization of SNF2h and hence chromatin opening and repair factor recruitment. PARylation of SNF2h in response to DNA damage (52) may reinforce NuMA–SNF2h interaction during the DDR. This model is in agreement with the observation that PARP activity promotes SNF2h accumulation at DNA breaks (42) and Figure 3g.

Additional mechanisms regulating SNF2h recruitment to sites of DNA damage enable us to envision the role of NuMA in the DDR as part of an integrative process. SNF2h association with the chromatin relies on histone marks, in particular H3K4 di- and trimethylation at actively transcribed loci and DNA breaks (7,54,55). NuMA silencing does not alter H3K4 trimethylation levels (unpublished results), suggesting that NuMA likely regulates SNF2h through a distinct mechanism. Additional control of SNF2h recruitment to DNA breaks relies on the sirtuin deacetylase SIRT6 (43). Although we do not exclude the possibility of a functional connection between SIRT6 and NuMA, the DDR functions of SIRT6 and NuMA are clearly distinct: SIRT6 is required for 53BP1 foci formation and NuMA is not. Hence, the regulation of SNF2h by a structural protein such as NuMA represents an added level of control of a chromatin remodeler.

SNF2h is a shared component of distinct chromatin remodeling complexes, several of which are implicated in genome maintenance (4). WSTF/BAZ1B, the SNF2h partner in the WICH complex, regulates γH2AX kinetics (34). WSTF associates with PCNA at replication foci (56) and since PCNA is rapidly mobilized at DNA breaks, we speculate that this DNA clamp may also participate in WSTF recruitment at DNA damage sites. Although we identified WSTF as a NuMA partner in pull-down experiments, NuMA knock-down impaired the accumulation at DNA breaks of SNF2h, but not of PCNA and WSTF, suggesting that SNF2h binding to WSTF-PCNA is necessary but not sufficient for SNF2h accumulation or retention at the chromatin. Incidentally, our data support the concept that chromatin remodeling complexes assemble at the chromatin rather than exist as preformed nucleoplasmic multiprotein entities. SNF2h also associates with ACF1/BAZ1A and with RSF1 to form the CHRAC and RSF1 complexes, respectively. Previous reports have implicated both ACF1 (6) and RSF1 (57,58) in HR DNA repair and it will be interesting to determine if the DDR functions of these remodelers depend on NuMA. The mechanism behind the choice to assemble one or the other of these mutually exclusive complexes remains to be understood, and it is possible that NuMA or other scaffolding elements participate in these molecular decisions based on the structural context of the nucleus.

The physiological relevance of NuMA and SNF2h interaction is demonstrated by the epistasis of both proteins in homology-dependent DNA repair. Literature search indicates that the level of enrichment for NuMA in chromatin after UV-induced DNA damage compares to that of SNF2h and WSTF, as well as other major DDR factors such as 53BP1 (59). Here we bring evidence that NuMA controls the chromatin response to DNA damage: (i) The dependency on NuMA for homology-directed repair is measurable in the chromatin context but not with extrachromosomal DNA; (ii) silencing NuMA alters γH2AX kinetics. The lack of retention of the mark in NuMA-silenced cells may result from defective ISWI function since the prolonged γH2AX response (but not initial H2AX phosphorylation at serine 139) depends on H2AX Y142 phosphorylation by the SNF2h partner WSTF (34); (iii) higher-order chromatin organization, captured with texture descriptor of DAPI images, diverges in NuMA knock-down compared to controls after IR exposure and (iv) decompaction of the chromatin adjacent to DSBs requires NuMA expression. We envision that NuMA-dependent targeting of chromatin remodelers may influence multiple genomic processes relying on ISWI activity, including chromatin replication (56,60).

With the identification of new regulators of chromatin remodeling, the control of these regulators and the possible consequences for cell behavior come into question. The plasticity of NuMA distribution has been documented during carcinogenesis and differentiation via extracellular matrix (ECM) signaling (19). Blocking ECM signals alters NuMA distribution in the nucleus and compromises DNA repair (18). Evidence also suggests that ECM factors regulate ATP-dependent chromatin remodeling (61,62). We envision that NuMA participates in the integration of extracellular cues by chromatin remodeling complexes. Cancer cells respond differently to ECM signals and often display decreased HR capabilities. Altered NuMA expression and localization in cancer cells (20,63,64) might jeopardize the integration of ECM signals and chromatin remodeling, hence serving a cancerous phenotype. In particular, it might contribute to lower DNA repair capabilities and increased genomic instability in tumors and/or favor DNA repair in cancer cells resistant to chemotherapy via the maintenance of their sensitivity to ECM signaling (65). The role of NuMA in chromatin remodeling and its functional significance during cancer progression deserve further investigation.

## SUPPLEMENTARY DATA

Supplementary Data are available at NAR Online.

SUPPLEMENTARY DATA
