# Supplementary material for: NuMA promotes homologous recombination repair by regulating the accumulation of the ISWI ATPase SNF2h at DNA breaks
Source: Nucleic Acids Res. 2014 Apr 20;42(10):6365–79. doi: 10.1093/nar/gku296 (PMC4041463; doi:10.1093/nar/gku296)
Supplement: SUPPLEMENTARY DATA [file supp_42_10_6365__index.html]

NuMA promotes homologous recombination repair by regulating the accumulation of the ISWI ATPase SNF2h at DNA breaks — NuMA promotes homologous recombination repair by regulating the accumulation of the ISWI ATPase SNF2h at DNA breaks — SUPPLEMENTARY DATA 

# NuMA promotes homologous recombination repair by regulating the accumulation of the ISWI ATPase SNF2h at DNA breaks

## SUPPLEMENTARY DATA

**Files in this Data Supplement:**

- SUPPLEMENTARY DATA
