# Supplementary material for: NuMA promotes homologous recombination repair by regulating the accumulation of the ISWI ATPase SNF2h at DNA breaks
Source: Nucleic Acids Res. 2014 Apr 20;42(10):6365–79. doi: 10.1093/nar/gku296 (PMC4041463; doi:10.1093/nar/gku296)
Supplement: SUPPLEMENTARY DATA [file supp_gku296_nar-00342-m-2014-File010.pdf]

SUPPLEMENTARY FIGURES AND LEGENDS

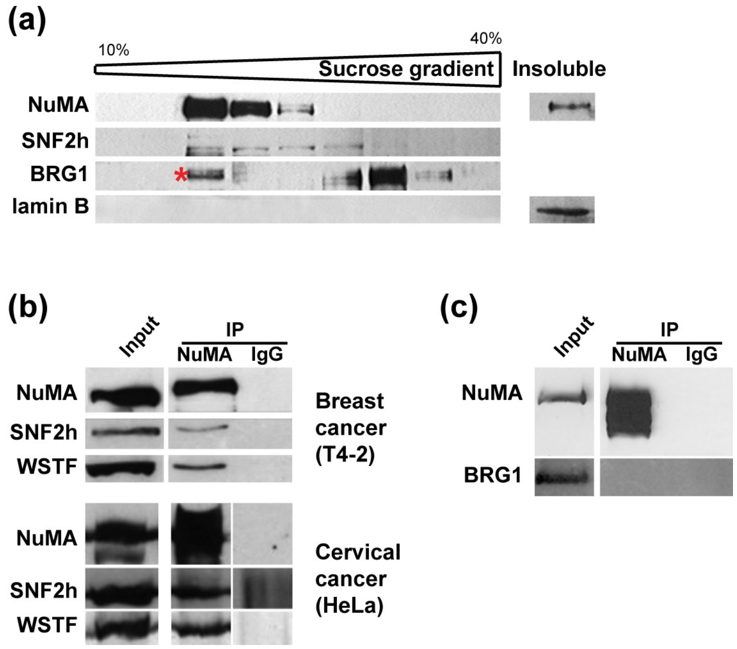

Figure S1. Interaction between NuMA and SNF2h. (a) Sucrose gradient fractionation of S1 nuclear extracts. Gradient fractions were analyzed by western blot for NuMA, ISWI ATPase SNF2h, SWI/SNF ATPase BRG1, and lamin B (a marker for insoluble nuclear proteins). The red asterisk indicates remaining signals from the NuMA immunoblot (shown on top) on the membrane subsequently reprobed with BRG1. (b) NuMA immunoprecipitation from T4-2 (top) and HeLa (bottom) nuclear extracts. Nonspecific IgGs were used as control and the blots were probed for NuMA, SNF2h, and WSTF. (c) NuMA immunoprecipitation from HeLa nuclear extracts probed with NuMA and BRG1 antibodies.

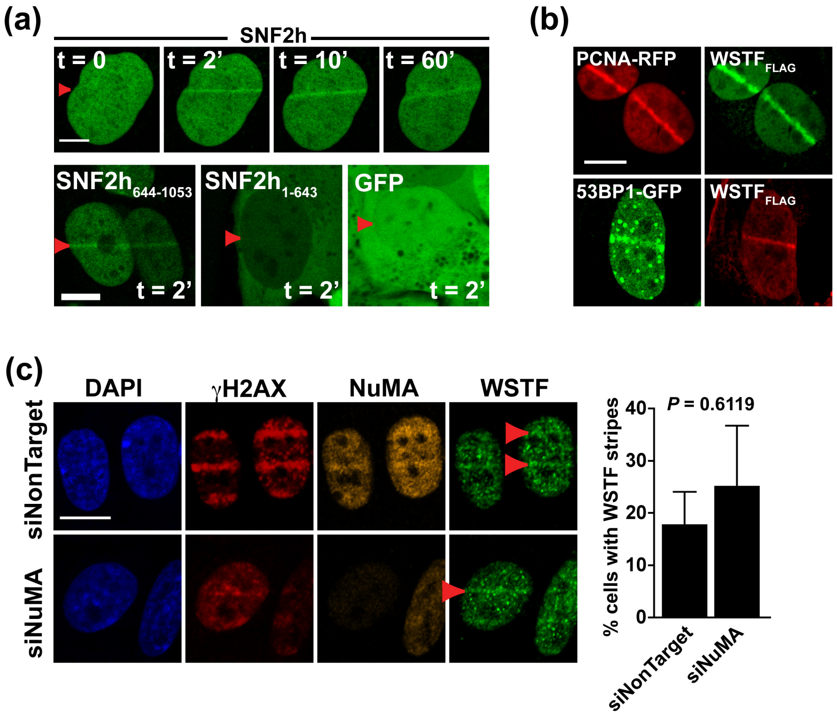

Figure S2. Accumulation of SNF2h and WSTF at sites of DNA damage. (a) Localization of full-length (top panel) and truncated (644-1053; 1-643) SNF2h-GFP in U2OS cells after laser microirradiation (arrowheads). GFP alone was used as control. Images were taken at the indicated time points. (b) WSTF immunostaining in microirradiated cells. Cells were cotransfected with FLAG-tagged WSTF and fluorescently tagged PCNA or 53BP1 to locate DNA damage sites. WSTF was detected using FLAG antibodies. (c) Endogenous WSTF at irradiation stripes. S1 cells treated with NuMA siRNA or with nontargeting siRNA were microirradiated and stained for γH2AΞ (to locate DNA damage), NuMA (to verify silencing) and WSTF. The bar graph represents the percentages of nuclei with overlapping WSTF and γH2AX stripes (*n* = 3 biological replicates). Scale bars, 10 μm

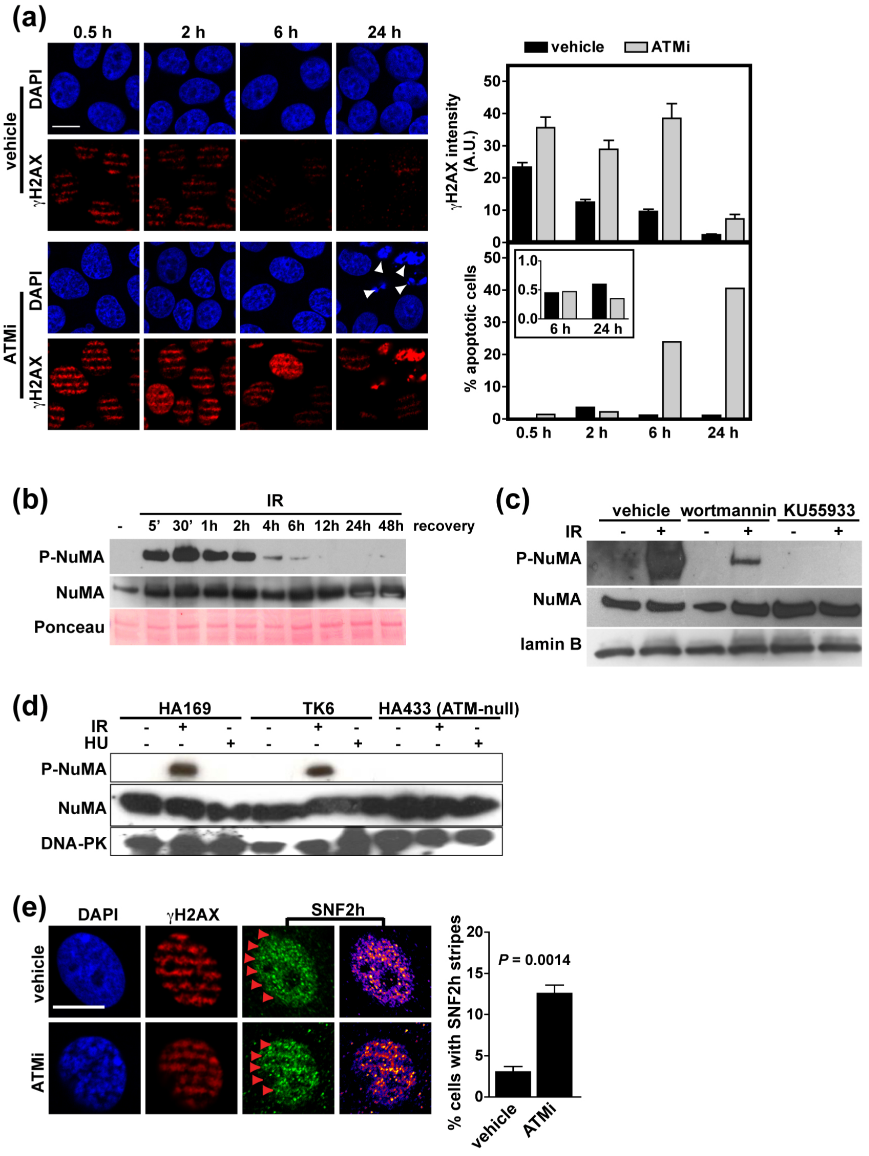

Figure S3. NuMA phosphorylation by ATM and effect of ATM inhibition on SNF2h accumulation at microirradiation sites and on microirradiation damage repair. (a) Repair of microirradiation-induced DNA damage in the absence and presence of the ATM-specific inhibitor KU55933 (ATMi). S1 cells were fixed and stained for γH2AX at the indicated time points after microirradiation. Representative images are shown in the left panels. Average γH2AX intensities in irradiated nuclei and the fraction of apoptotic nuclei (condensed DAPI staining, arrowheads) are shown in the graphs. The inset in the bar graph with quantification of apoptosis in nonirradiated cells indicates lack of toxicity of the ATMi treatment (b-d) NuMA phosphorylation on serine 395 (P-NuMA) in response to DNA damage. (b) Western blot analysis of P-NuMA in MCF-7 cells exposed to gamma irradiation (IR, 3 Gy) and incubated for recovery as indicated. NuMA immunoblot and the Ponceau stain are shown as controls. (c) Cells treated for 1 hour with wortmannin (a pan-PI3K inhibitor), KU55933 (an ATM-specific inhibitor) or vehicle, were

irradiated, incubated for 30 minutes, and processed for P-NuMA, NuMA, and lamin B (loading control) immunoblots. (d) Lymphoblastoid cell lines expressing wild-type ATM (HA169 and TK6) or an ATM-null mutation (HA433) were irradiated (3 Gy) to activate ATM, or treated with hydroxyurea (HU; 1 hour, 1 mM) to activate ATR before analysis by western blot. The blot was probed with antibodies against P-NuMA, NuMA, and DNA-PK (loading control). (e) Immunostaining for SNF2h and γH2AX in microirradiated S1 cells treated with vehicle or ATMi. Representative images are shown (left). The percentage of cells with SNF2h accumulation at irradiation stripes is quantified (right; n = 3 biological replicates). Note that reduced laser intensity was used to induce DNA damage compared to Figure 2c in order to better quantify the increased recruitment of SNF2h upon ATMi treatment. Scale bars, 10 μm

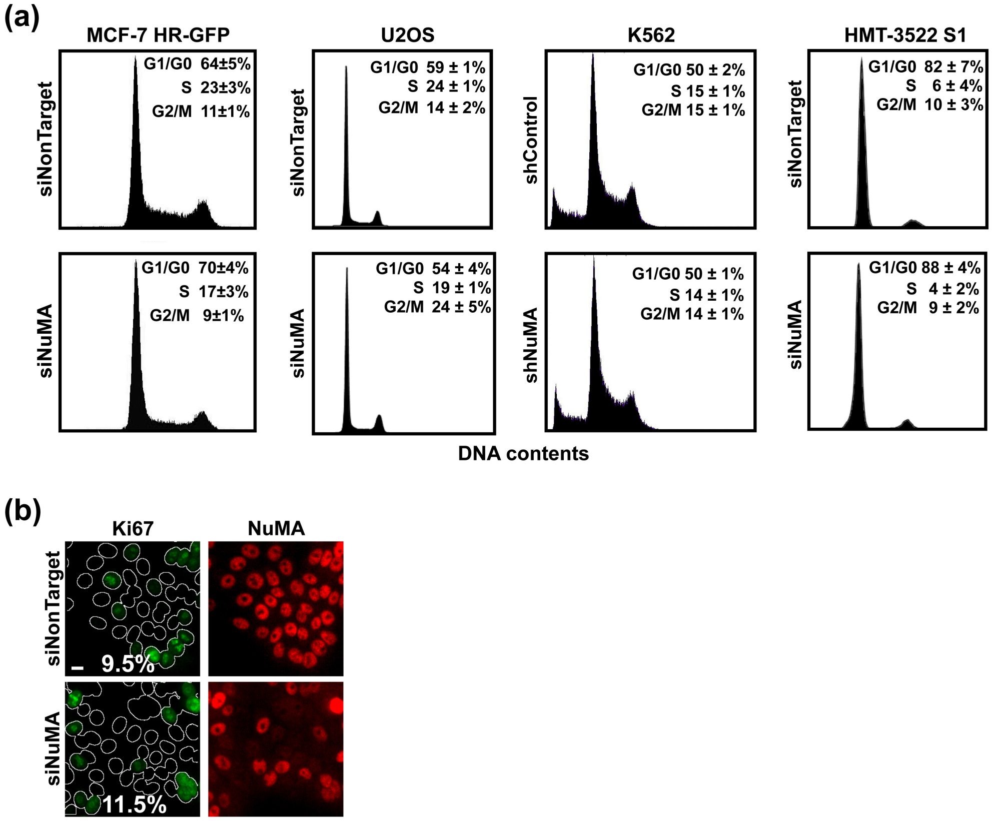

(c)

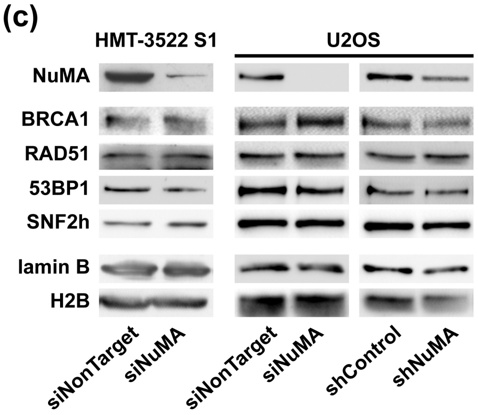

Relative band intensities (siNuMA/siNonTarget)

	HMT-3522 S1	U2OS (siRNA)	U2OS (shRNA)
NuMA	0.31 ± 0.08	0.06 ± 0.03	0.25 ± 0.10
BRCA1	N.D.	1.35 ± 0.36	1.10 ± 0.16
RAD51	1.41 ± 0.26	1.01 ± 0.08	1.09 ± 0.19
53BP1	1.42 ± 0.45	1.02 ± 0.26	1.02 ± 0.14
SNF2h	1.21 ± 0.26	1.18 ± 0.09	1.22 ± 0.34

Figure S4. Cell cycle distribution and expression levels of DDR factors upon NuMA silencing. (a) Cell cycle distribution analyzed by flow cytometry in MCF-7 HR-GFP, U2OS, K562, and S1 cells treated with siRNA or shRNA specific to NuMA or nontargeting. Values are mean ± SEM from at least three experiments. (b) Immunostaining for the Ki67 proliferation marker in S1 cells treated with NuMA siRNA or with nontargeting siRNA. Boundaries of the cell nuclei are indicated on the Ki67 images. The percentages of Ki67 positive cells are indicated. Scale bar, 10 µm. (c) Western blot analysis of NuMA, BRCA1, RAD51, 53BP1, and SNF2h expression in S1 and U2OS cells transfected with NuMA-specific or nontargeting si- and shRNA, as indicated. Lamin B and H2B were used as loading controls. Signal quantification by densitometry (NuMA-silenced relative to control; $n \geq 3$) is shown in the table.

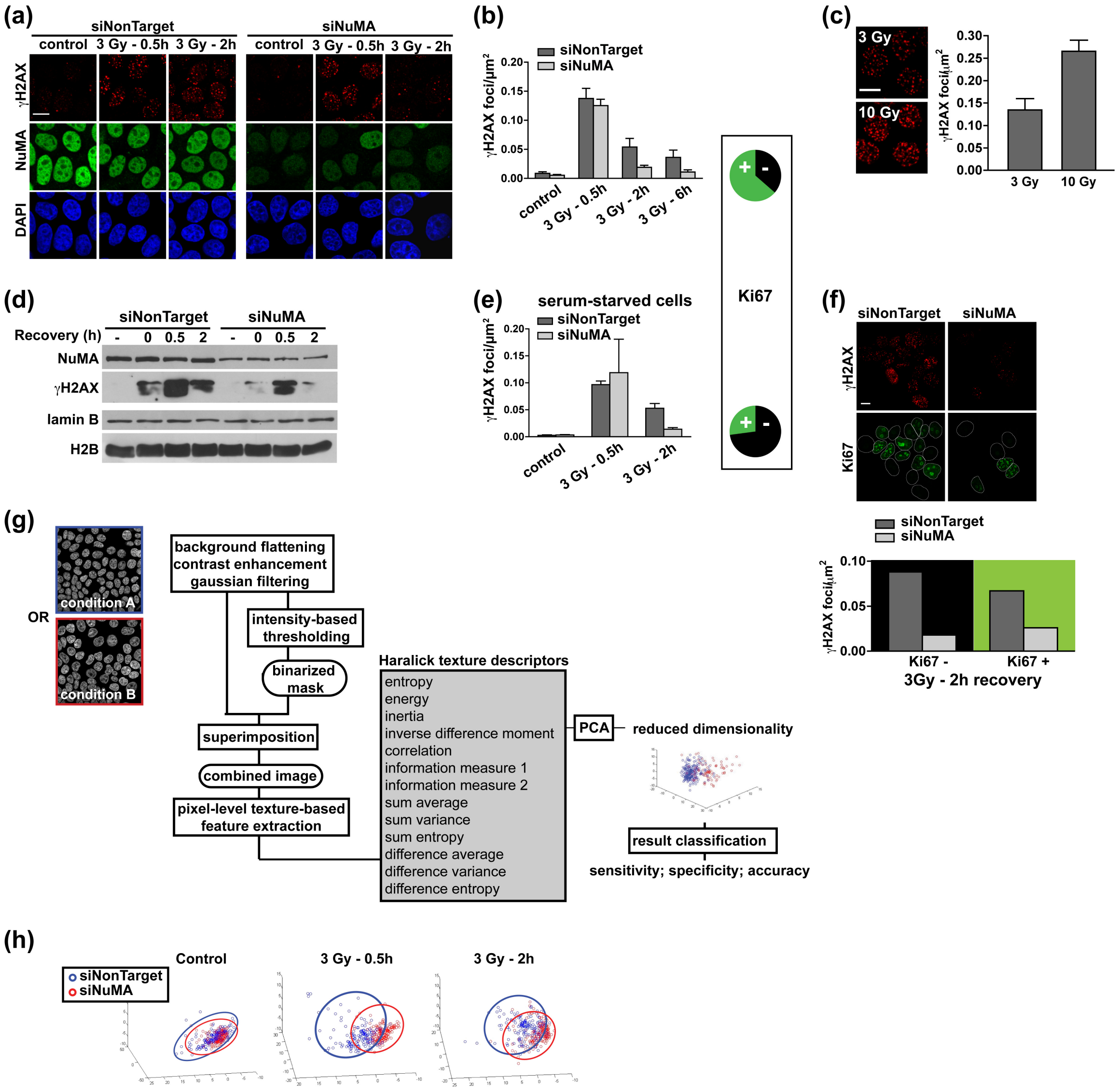

sensitivity	58	80	75
specificity	74	94	79
accuracy	0.66	0.87	0.77

Figure S5. H2AX phosphorylation and chromatin response in cells silencing NuMA. (a) Confocal images of MCF-7 cells transfected with NuMA siRNA or with nontargeting siRNA and stained for γH2AX and NuMA after irradiation (IR, 3 Gy) at the times indicated. (b) γH2AX foci density quantified in confocal images as shown in A. Values normalized to siNonTarget are shown (inset; *n* = 3; * *P* < 0.05, one-sample t-test). (c) Representative γH2AX patterns and quantification of foci density in cells exposed to 3 and 10 Gy of ionizing radiations. (d) Western blot analysis of cells treated as in A, using antibodies against γH2AX, NuMA (to assess silencing), as well as lamin B and H2B (loading controls). (e) γH2AX foci density in MCF-7 cells treated as in A-B, but with serum omitted from the culture medium 24 hours before IR. Reduced cell proliferation after serum starvation is evidenced by Ki67 scores (pie charts). (f) Quantification of γH2AX foci as in B, but guided by the cell proliferation status (Ki67 signals). Shown are representative images of γH2AX and Ki67 immunostainings (lines indicate the boundaries of nuclei) and graphs for γH2AX

quantification. (g) Schematic of the high-content analysis of texture descriptors. PCA, principal component analysis. (h) Chromatin texture (DAPI-stained nuclei) analyzed in MCF-7 cells transfected with NuMA siRNA or with nontargeting siRNA. Cells were irradiated (IR; 3 Gy) and left to recover as indicated. Sensitivity, specificity and accuracy quantify parting of cell populations. Scale bars, 10 μm
